# UEG Journal's impact and vision for the future

**DOI:** 10.1002/ueg2.12535

**Published:** 2024-01-27

**Authors:** Omar Elshaarawy, Alberto Balduzzi, Anna Burelli, Zsa Zsa R. M. Weerts

**Affiliations:** ^1^ Department of Gastroenterology University of Liverpool Royal Liverpool University Hospital Liverpool UK; ^2^ Department of Gastroenterology and Hepatology National Liver Institute, Menoufia University Shebine Elkom Egypt; ^3^ Department of Surgery, Dentistry, Paediatrics and Gynaecology Unit of General and Pancreatic Surgery The Pancreas Institute Verona University of Verona Verona Italy; ^4^ General Surgery Unit IRCCS Sacro Cuore Don Calabria Hospital Negrar di Valpolicella Verona Italy; ^5^ Division Gastroenterology‐Hepatology Maastricht University Medical Center+ Maastricht The Netherlands

**Keywords:** AI, endoscopy, gastroenterology, hepatology, oncology, pancreas, screening

## INTRODUCTION

The UEG Journal has become a leading international journal in gastroenterology during the past years and aims to be in the top 15% of gastroenterology journals worldwide, serving as a well‐known forum for research on digestive diseases. We feel that this vision has come to pass as we commemorate the journal's 10th anniversary. Over these 10 years, The UEG Journal has received over 50,000 submissions and identified and published significant scientific advancements with a real impact on clinical practice. The journal's growing citation metrics—which include an astounding impact factor of 6 and more than 18,000 total citations—indicate that the gastro intestinal community is paying attention. The commitment of our reviewer community, trainee editors, editors, and readers all contribute to the UEG journal's success.

Through its online open‐access format, podcasts, social media broadcasts, and visual abstracts, the UEG Journal has become even more appealing. The Journal is fully dedicated to maintaining high standards for the calibre of research as we move into 2024. We are always keeping a close look out for future advancements; three areas that we intend to concentrate on are automation, artificial intelligence, and the gut microbiome.

## REFLECTING ON 2023

The inflammatory bowel disease (IBD) field, our Journal provided unique real‐world data confirming that the benefits of a controlled disease outgrow the potential adverse effects of anti‐TNF alfa antibodies during pregnancy.^1^ In this large cohort of 293 pregnancies from the post‐marketing PYRAMID registry, adalimumab did not impair pregnancy outcomes adding an important piece of information in normalizing pregnancy in women with Crohn's disease.

Two papers published this year were focused on cancer prevention, one investigating the cost‐effectiveness of surveillance for pancreatic ductal adenocarcinoma (PDAC) in CDKN2A‐p16‐Leiden mutation carriers and the other proposing real‐time artificial intelligence (AI)‐assisted colonoscopy for detecting colorectal cancer in Lynch Syndrome patients.^2^, ^3^ In the Netherlands, a 20‐year surveillance program in CDKN2A‐p16‐Leiden mutation carriers led to early detection of PDAC with improved resectability and survival.^4^ However, the increasing economic burden that public health systems face nowadays may prompt worries about costs. This paper showed that annual MRI and optional endoscopic ultrasound is cost‐effective in patients at risk of pancreatic cancer with an estimated cost of around €14000 ($15345) per quality‐adjusted life year (QALY), well below the $50000 per QALY threshold.^5^

On the other hand, the CADEYE study, a pilot randomized trial, revealed real‐time AI‐assisted colonoscopy as a promising tool to optimize endoscopic surveillance in Lynch Syndrome patients compared to conventional colonoscopy, with similar examination times and higher detection rates of flat adenomas.^3^ As recently stated by the World Health Organization, AI has the potential to “enhance health outcomes by strengthening clinical trials, improving medical diagnosis, treatment, self‐care and patient‐centred care, and supplementing health care professionals' knowledge, skills and competencies”. We, as the UEG Journal community, are strongly involved in contributing to reporting evidence on this promising tool. This paper rightfully received accolades as the UEG Journal Paper of The Year.

## GUIDELINES MOVE CLINICAL CARE

The most reliable framework for synthesizing evidence is still clinical practice guidelines, which steer clinicians toward the best possible treatment. Eighteen guidelines on the diagnosis and treatment of digestive health issues have been published by the UEG Journal since 2013. We continue to be committed to publication of these guidelines for the years to come.

To improve the usability and accessibility of guidelines, several features have now been included, such as visual abstracts and algorithms. In addition, the UEG Guideline app has been created, providing clinicians with simple access to 39 guidelines and 130 interactive tools, making it easier for recommendations to be implemented in clinical practice.^6^, ^7^

## PODCASTS: GASTROENTEROLOGICAL INSIGHT RIGHT TO YOUR EARS

As we delve further into the dynamics of our communication landscape, the UEG Journal podcasts emerge as a unique experience. Our audience has access now to more than 24 episodes, around 7 h of recordings, discussing behind the scenes stories covering exceptional manuscripts form all aspects of gastroenterology. These podcasts offer a conversational and accessible format for our audience. Through insightful discussions with key contributors, our podcasts aim to amplify the narrative, making complex topics more approachable and fostering a sense of connection within our UEG community.

## CRAFTING A DIGITAL NARRATIVE OF ENGAGEMENT: SOCIAL MEDIA AND VISUAL ABSTRACTS

In order to connect with our community, the Editorial Board has expanded its efforts beyond The UEG Journal pages and now uses Social Media platforms such as *X* (previously Twitter) a means of communication. UEG journal has harnessed the power of platforms like *X*, boasting a commendable milestone of 10,000 followers on X. This accomplishment signifies the reach of the UEG Journal with the community.

Through visual abstracts created by our trainee editors and Susan Tyler, our illustrator, we translate the traditional scientific language text into compelling graphics with a clear message that captures the attention on social media. We hope that this resonates with clinicians, researchers, and enthusiasts alike.

For example, two visual abstracts on IBD manuscripts have garnered more than 13,000 views.^8^, ^9^ This is a great engagement not only amplified the reach of our content, but also provided an invaluable platform for knowledge exchange and collective learning.

A relatively new section of the journal is that of the clinical image. In 2023, we have published 8 clinical images that offer educational quick wins by showcasing rare findings combined with a short informative, and accessible text.

## CHARTING OUR COURSE

In 2023, a dynamic team of 15 enthusiastic trainee editors joined forces to elevate the UEG Journal.^10^ This editorial squad is a powerhouse, segmented into specialized groups—skilful reviewers in fields like IBD, hepatology, oncology, luminal, neurogastroenterology, pancreas, and endoscopy. The trainee editors’ contributions and enthusiasm is the fuel of our podcasts, the clinical images series, the social media engagement, and the promotion of UEG Journal. In addition, they perform peer review contributing to the quality of the UEG Journal.

As we navigate the uncharted waters of 2024, the UEG Journal is guided by emerging priorities *s*. One of the most anticipated landmarks are the UEG Liver Diseases and Pancreas Diseases Special Issues, set to be published in 2024 and will bring together the latest research, innovative treatments, and comprehensive reviews from international authors, providing a valuable resource for the new year.

Soon, the UEG guidelines task force, led by Yasuko Maeda our associate editor, will report on the quality of available guidelines. The taskforce is currently planning a survey about guidelines, targeting both clinicians and patients, reflecting their experiences and needs. The outcomes of the survey will be pivotal in identifying areas for improvement and developing solutions to enhance the quality of our recommendations.

Finally, as we chart our course for 2024, we are excited about the journey ahead and the opportunities it presents.

## Data Availability

Data openly available in a public repository that issues datasets with DOIs.
